# Draft Genome Sequence of Permafrost Bacterium *Nesterenkonia* sp. Strain PF2B19, Revealing a Cold Adaptation Strategy and Diverse Biotechnological Potential

**DOI:** 10.1128/genomeA.00133-17

**Published:** 2017-04-13

**Authors:** Purnima Singh, Neelam Kapse, Utpal Roy, Shiv Mohan Singh, P. K. Dhakephalkar

**Affiliations:** a Birla Institute of Technology and Science (BITS), Pilani-K.K. Birla Goa Campus, Zuarinagar, Goa, India; b Agharkar Research Institute (ARI), Maharashtra Association for Cultivation of Science, Pune, India; c National Centre for Antarctic and Ocean Research (NCAOR), Ministry of Earth Sciences, Vasco-Da-Gama, Goa, India

## Abstract

*Nesterenkonia* sp. strain PF2B19, a psychrophilic bacterium, was isolated from 44,800-year-old permafrost. The draft genome sequence of this strain revealed the presence of genes involved in the production of cold active enzymes, carotenoid biosynthesis, fatty acid biosynthesis, and resistance to heavy metals. These results show the immense potential of the strain.

## GENOME ANNOUNCEMENT

Permafrost soils are chronological archives of microorganisms (1). Microbial life has survived in a metabolically active state in the Svalbard permafrost (2–6). PF2B19, a Gram-positive coccus and aerobic bacterium, was isolated from permafrost (44,800 years old) in Svalbard. In order to unravel the molecular mechanisms underlying the cold adaptation and to identify biotechnologically relevant cold-adapted enzymes, whole-genome sequencing was performed.

The genome of *Nesterenkonia* sp. strain PF2B19 was sequenced using 316 Chip and 200-bp chemistry on the Ion Torrent PGM platform (Life Technologies, Inc., USA), which generated 2,662,620 bp of reads. The reads were *de novo* assembled using MIRA assembler version 4.0.5 (7) into 2,924 contigs, yielding a genome of ~2.6 Mb in size, with a G+C content of 67.6%. These results are similar to the sizes (2.59 to 2.81 Mb) and G+C contents (62.2 to 71.5%) detected in the draft genomes of three strains of *Nesterenkonia*, available in the public databases. The genome was annotated using the Rapid Annotations using Subsystems Technology (RAST) server (8). A total of 3,482 proteins were predicted, including 3,434 coding sequences and 47 total RNAs (nine rRNAs and 38 tRNAs). Digital DNA-DNA hybridization, performed as described by Auch et al. (9), revealed only 30.20%, 26.50%, and 27.40% homology between PF2B19 and *Nesterenkonia* JCM 19054, *Nesterenkonia alba* DSM 19423^T^, and *Nesterenkonia* sp. strain AN1, respectively, indicating a distinct delineation between the species and also depicting the novelty of strain PF2B19.

Further, the genome of PF2B19 was compared with the available *Nesterenkonia* genomes, which generated the nucleotide alignments of the genomes by BLASTn using the PF2B19 as the reference and the program BRIG (10). BLASTn results of each genome (*Nesterenkonia* JCM 19054, *Nesterenkonia alba* DSM 19423^T^, and *Nesterenkonia* sp. AN1) against PF2B19, with results rendered using the BRIG program revealed pronounced gaps in the query genomes highlighting the distinction between PF2B19 and the other *Nesterenkonia* genomes used.

The Arctic is characterized by harsh cold conditions. A number of genes linked to cold adaptation have been reported in the literature (11–14). Analysis of the draft genome of *Nesterenkonia* sp. PF2B19 revealed multiple genes encoding a repertoire of proteins (a total of 78 genes) associated with cold stress adaptive response. These included cold shock proteins CspA and CspC (nine genes); genes encoding oxidative stress alleviating enzymes namely catalase (24 genes); superoxide dismutases (SodA and SodC- two genes each), a thiol peroxidase (Bcp), thioredoxin and thioredoxin reductase (TrxA and TrxB). Thirty two genes encoding osmotic stress response, transporters for glycine/betaine and other compatible solutes, and choline dehydrogenases were also detected.

Analysis of the annotated genome sequence of PF2B19 revealed the presence of genes involved in the production of α-amylases, including maltase and maltodextrinase, as well as xylanase, highlighting its biotechnological potential.

Thus, by means of genomic analyses, we could elucidate the genetic determinants of the adaptive strategies employed by *Nesterenkonia* sp. PF2B19 for survival in the cold permafrost soils of the Arctic.

### Accession number(s).

The whole-genome shotgun project has been deposited in DDBJ/EMBL/GenBank under accession number MDSS00000000.
